# Barriers and facilitators for smoking cessation in chronic smokers with atherosclerotic cardiovascular disease enrolled in a randomized intervention trial: A qualitative study

**DOI:** 10.3389/fpsyg.2023.1060701

**Published:** 2023-03-22

**Authors:** Vilde Getz, John Munkhaugen, Hanne C. Lie, Toril Dammen

**Affiliations:** ^1^Faculty of Medicine, Institute of Clinical Medicine, University of Oslo, Oslo, Norway; ^2^Department of Behavioural Medicine, Faculty of Medicine, Institute of Basic Medical Sciences, University of Oslo, Oslo, Norway; ^3^Department of Medicine, Drammen Hospital, Drammen, Norway; ^4^Division of Mental Health and Addiction, Oslo University Hospital, Oslo, Norway

**Keywords:** smoking cessation, qualitative, facilitators and barriers, cardiovascular disease, secondary prevention

## Abstract

**Objectives:**

Smoking is common in patients with cardiovascular disease. Despite strong recommendations for cessation and the existence of efficacious pharmacological and behavioral interventions, cessation rates remain low. Therefore, in this study, we explore perceived facilitators and barriers to smoking cessation in patients with atherosclerotic cardiovascular disease who have participated in a cessation intervention study.

**Methods:**

Participants (*N* = 10) from the intervention arm of a randomized controlled study with access to free cessation support and pharmacological aids completed a semi-structured, in-depth telephone interview after a 6-monthfollow-up between October 2021 and July 2022. The interviews were audio recorded, transcribed, and analyzed according to principles of thematic analysis.

**Results:**

The mean age was 65.7 (range: 55–79) years, and three of the 10 participants were women. Among the participants, five had quit smoking, three had relapsed, and two were persistent smokers. The themes identified encompassed barriers and facilitators to cessation, both including individual and contextual factors. Barriers included the upsides of smoking, difficult life situations, addiction to smoking, smoking in social circles, perceived lack of support and understanding from health professionals. Facilitators included intrinsic motivation, concerns about the health condition, financial implications, specific behavioral strategies, positive influence from the social environment, and helpful components of the cessation intervention.

**Conclusion:**

Smokers with cardiovascular disease who have attended a cessation intervention study report several facilitators weighted against barriers, interacting with the intention to cease smoking. The most important potentially modifiable factors of significance for cessation identified may be addressed through motivational interviews and focus groups with other smokers.

## 1. Introduction

Smoking is among the modifiable risk factors for non-communicable diseases worldwide (Doll et al., 2004; National Center for Chronic Disease Prevention Health Promotion (US) Office on Smoking Health, 2014). Of those dying as a consequence of smoking, it is estimated that half of the deaths are due to cardiovascular disease (CVD) (Visseren et al., 2021). It is, therefore, concerning that the proportion of daily smokers among patients with CVD in Europe has only decreased from 20 to 16% over the past 20 years (Kotseva et al., 2009, 2016). This is a slight decline in the rates of smoking among patients with CVD, but it is very narrow, and the rates remain high. Moreover, recent Norwegian data have shown that 28–36% of patients admitted to hospital with an acute CVD event were daily smokers (Sverre et al., 2017; Kaldal et al., 2021). Similar rates have been reported in a large European study (Kotseva et al., 2016).

Smoking cessation is potentially the most effective lifestyle measure to prevent future cardiovascular events (Critchley and Capewell, 2004; Edwards, 2004; Visseren et al., 2021). A meta-analysis found a relative risk reduction for coronary mortality of 46% (Wilson et al., 2000), whereas a systematic review found a decrease in all-cause mortality of 36% in those who stopped smoking compared to those who continued to smoke (Critchley and Capewell, 2004). This reduction in mortality appears to be just as effective compared to other secondary preventive drugs, such as cholesterol-lowering and antihypertensive drugs (Critchley and Capewell, 2004). Thus, smoking cessation is recommended with the highest level of evidence in both national and international guidelines for the prevention of CVD (Helsedirektoratet, 2017; Visseren et al., 2021). In particular, effective behavioral interventions and pharmacological treatments for facilitating cessation exist. According to a Cochrane review of randomized studies involving pharmacological cessation aids, varenicline significantly increased the likelihood of quitting smoking compared with a placebo. Moreover, a combination of long- and short-acting nicotine replacement therapy (NRT) was found to be as effective as using varenicline (Cahill et al., 2013). Hospitalization for an acute CVD event is recognized as an important opportunity to facilitate smoking cessation, and intensive counseling with or without motivational interview techniques may increase cessation rates by 65% (Rigotti et al., 2012). Most effective is a combination of cessation drugs and behavioral support, strongly recommended for patients with CVD (Hartmann-Boyce et al., 2014, 2019; Visseren et al., 2021). Nevertheless, the rate of successful cessation in clinical practice is relatively poor. In a large European study, Kotseva et al. found that ~50% of patients quit smoking after an acute CVD event (Kotseva et al., 2016). One of the reasons for poor cessation rates could be the limited empirical evidence on how to incorporate effective smoking cessation interventions into routine clinical practice (Rigotti et al., 2012; Visseren et al., 2021).

Several barriers to smoking cessation have been identified. As smoking is highly addictive through nicotine dependency, a daily smoker will need a regular supply of nicotine to avoid unpleasant withdrawal symptoms and to achieve the pleasant effects nicotine causes through stimulation of the reward center (Benowitz, 2010). Several aspects can influence biological dependence, such as psychological and environmental conditions (Shadel et al., 2000; Pfeffer et al., 2018). Research has suggested that the smoking population today is largely an underserved group characterized by low socioeconomic status and with limited personal resources to facilitate cessation (Cavelaars et al., 2000; Laaksonen et al., 2005; Hiscock et al., 2012). Other known barriers include, but are not limited to, psychological factors, lack of support, nicotine dependency, lack of motivation, low adherence to treatment, and stress (Hiscock et al., 2012). A Norwegian study found associations between persistent smoking after a cardiovascular event and unemployment/disability benefits, low education, and long smoking duration (Sverre et al., 2017).

Qualitative studies exploring smokers' facilitators and barriers, motivation, and experiences with smoking cessation have also been conducted (Kerr et al., 2006, 2013; Medbø et al., 2011; Clancy et al., 2013; Buczkowski et al., 2014, 2021; Boland et al., 2017). These have either included smokers because of certain smoking characteristics, such as previously unsuccessful quit attempts (Buczkowski et al., 2014, 2021), or specific groups, e.g., people with psychological issues (Clancy et al., 2013; Kerr et al., 2013), low socioeconomic status (Boland et al., 2017), or elderly above 60 years (Kerr et al., 2006; Medbø et al., 2011). Our literature review has not identified any qualitative studies investigating the perceptions of smokers who have recently been hospitalized with an acute CVD event. This is important because these patients are at particularly high risk for a poor cardiovascular prognosis if they continue smoking (Prescott et al., 1998; National Center for Chronic Disease Prevention Health Promotion (US) Office on Smoking Health, 2014; Visseren et al., 2021). Furthermore, we are not aware of any studies including patients who have been offered participation in an intervention study with access to recommended cessation management including free cessation aids and close follow-up care (Visseren et al., 2021). Such knowledge will broaden our understanding of the mechanisms underpinning successful smoking cessation and be useful for informing effective cessation interventions for this subgroup of smokers. Our aim was, therefore, to explore perceived facilitators and barriers to smoking cessation among smokers with CVD who had been offered participation in a cessation program.

## 2. Material and methods

### 2.1. Study design, population, and ethics

This is a planned, qualitative individual, semi-structured interview study of patients hospitalized with an acute CVD event who participated in an open-label, single-center, and randomized controlled trial (RCT) (ClinicalTrials.gov, Identifier: NCT04772144). The inclusion criteria were age ≥18 years, smoking at least one cigarette daily when admitted to the hospital with an acute atherosclerotic CVD event (i.e., myocardial infarction, angina pectoris, carotid stenosis, or claudication in need of revascularization). The exclusion criteria were chronic kidney disease stage four, known contraindications to varenicline, any condition (e.g., psychosis, alcohol abuse, and dementia) or situation that may pose a significant risk to the participant, confound the results, or make participation unethical, short life expectancy (< 12 months), or lack of Norwegian language skills.

Participants were randomized to either an intervention group or a control group. The intensive intervention included a structured nurse-led in-hospital intervention focusing on motivation for cessation by utilizing motivational interview techniques. Participants also received detailed information about cessation drugs. They were referred to a 12-week digital or face-to-face cessation program at the municipal Healthy Life Center (HLC) with access to pharmacological drug treatment free of charge. To evaluate the intervention and to identify future intervention targets, only participants randomized to the intervention group in the randomized study were recruited for the qualitative study. The primary outcome in the RCT was self-reported smoking status at the 6-month follow-up. Abstinence was verified objectively with measurements of carbon monoxide in exhaled air.

The study was conducted in accordance with the ethical principles of the Declaration of Helsinki and in consistence with ICH/Good Clinical Practice. The study protocol, including the qualitative interviews, was reviewed by the Regional Committee for Medical and Health Research Ethics without remarks (202686) and approved by the local Data Protection Officer (21-0048-1). All participants provided written informed consent.

### 2.2. Recruitment to the qualitative study

Participants were consecutively recruited at the 6-month follow-up of the randomized clinical study, after data collection, by a study nurse. Of 27 patients included in the RCT, a total of 25 patients were invited to participate, of whom 10 participants consented and completed the interview (40% response rate).

### 2.3. Clinical and psychological characteristics

Patient characteristics were obtained from hospital medical records and validated questionnaires were completed at the time of randomization. In this sub-study, we used information on age, gender, CVD history, marital status, and education as background descriptions. In addition, we included data on smoking history and degree of nicotine addiction using the Fagerström Test for Nicotine Dependence (Heatherton et al., 1991).

### 2.4. Interviews

The interviews were conducted by the first author, a medical student who was trained and supervised by two experienced qualitative researchers (TD, HCL). The interview lasted for 45–90 min and focused on the patient's experience with the cessation process. The interviews were conducted over the telephone 5–9 months after hospitalization, audio recorded, transcribed ad verbatim, and de-identified. A semi-structured interview guide was used to explore facilitators and barriers associated with smoking cessation and experiences with the intervention (cessation aids, motivational interview techniques, and the HLC program). The interview guide was informed by a literature review and the experience of an interdisciplinary clinical research group with extensive clinical experience in preventive cardiology and risk-behavior change, including smoking cessation (refer to themes explored in Table 1 and Supplementary material). All transcripts were reviewed and discussed by the interdisciplinary group with competency in preventive cardiology (JM), behavioral medicine and psychiatry (TD), and health psychology (HCL). Recruitment continued until information saturation was considered to be reached, that is, when no new themes arose in three subsequent interviews.

**Table 1:** Questions used in the semi-structured interview guide.

**Explorative themes**
- Reasons for having succeeded or not in quitting smoking
- Decisive factors for having succeeded or not in quitting smoking
- Factors strengthening/decreasing motivation to quit smoking
- Attendance at the cessation program at the HLC
- Use of cessation drugs
- Perceived importance of the cessation follow-up for the outcome
- Experiences from the received smoking support

### 2.5. Analysis

The interviews were analyzed using a deductive–inductive adaptation of Braun and Clarke's six-stage method for thematic analysis (Braun and Clarke, 2006). NVivo 12 software was used to organize and code the data. First, the transcripts were read several times to familiarize with the content and to form an overall impression of the material. Second, initial codes were deductively created based on statements that could be placed under “barriers to cessation” or “facilitators to cessation,” which later became the overarching themes. Third, within the barriers and the facilitator themes, codes were inductively developed and then grouped into themes covering motivational, behavioral, psychological, social, and treatment factors. Fourth, these themes were reviewed several times for grouping and merging, resulting in sub-themes divided into individual and contextual factors. Fifth, the essence of each sub-theme was defined, and describing names were given. Finally, the report was written. The data analysis was carried out by VG, who had regular contact with TD throughout the analysis process to discuss and redefine themes and sub-themes. These were then discussed several times with JM and HCL. All co-authors have read all the transcripts and participated in several discussions before the final categorizations were made in the final stage. Supporting quotes are presented in the Results Section.

## 3. Results

In total, 10 patients were included, three women and seven men. Mean age was 65.7 (range: 55–79) years. One participant had education beyond high school. Six lived with a partner, of whom five smoked too. All but one of the participants were ethnically Norwegian. Further clinical characteristics are presented in Table 2.

**Table 2:** Patient characteristics.

**Participant name^a^**	**Sex**	**Smoking status^b^**	**Smoking duration (years)**	**Level of nicotine addiction^c^**	**Motivation to quit^d^**	**Previous quit attempt**	**Participated in HLC**	**Used pharmacological cessation aids**
Henrik	Male	A	>40	3	8	Yes	Yes	Yes
Lisa	Female	A	>40	3	7	Yes	Yes	Yes
Thomas	Male	A	21–30	5	10	Yes	No	No
Anna	Female	A	>40	3	10	Yes	No	No
Emilia	Female	A	21–30	3	7	No	No	No
Hans	Male	R	11–20	4	6	No	No	No
Peter	Male	R	31–40	4	10	No	Yes	Yes
Mike	Male	R	31–40	4	10	Yes	Yes	Yes
Oscar	Male	S	11–20	1	10	Yes	No	No
Kristian	Male	S	>40	6	7	No	Yes	Yes

a Pseudonyms by the first author.

b A, abstinent from smoke; R, relapse, defined as smoking after a period of at least 24 h of continuous non-smoking between randomization and follow-up; S, smoker.

c Level of nicotine addiction on a scale from 0 to 10, where 0–2 is very low dependence, 3–4 is low dependence, 5 is medium dependence, 6–7 is high dependence, and 8–10 is very high dependence (Heatherton et al., 1991).

d Motivation to quit on a 0 (no motivation) to 10 (high motivation) Likert scale.

**Figure 1:**
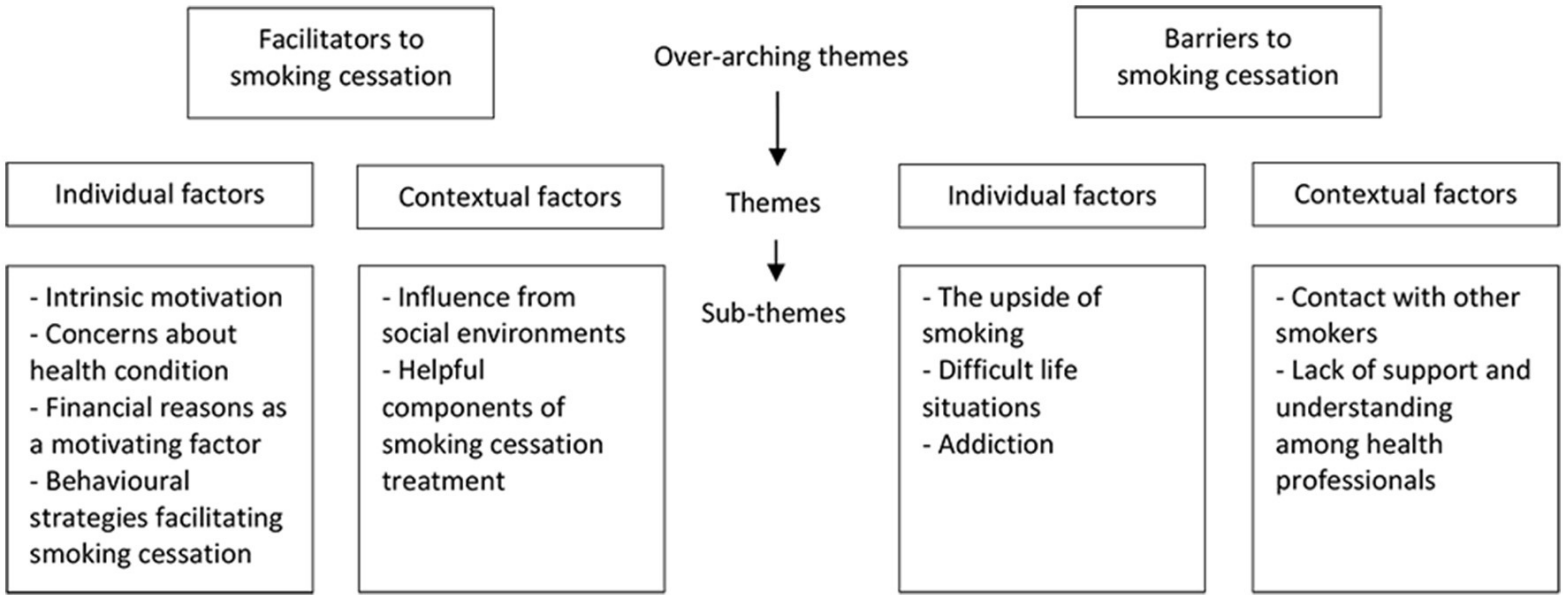
Overview of themes and sub-themes developed in the thematic analysis.

Two overarching themes were identified, facilitators and barriers to cessation, covering both individual and contextual factors and encompassing six and five sub-themes, respectively, as shown in Figure 1.

When presenting the results, pseudonyms are used, followed by smoking status, to identify the quotes, i.e., A (abstinent from smoking), R (relapsed to smoking), and S (never stopped smoking). The marker “//” indicates that some sentences were left out.

### 3.1. Facilitators of smoking cessation

#### 3.1.1. Individual factors

##### 3.1.1.1. Intrinsic motivation

Participants described a range of individual factors important for facilitating cessation. Successful cessation was seen to depend on their own willpower, and it was ultimately up to them to decide to quit smoking. That is, no one could force them or pressure them to quit:

Kristian, S: “I know that there is one person who can make me stop smoking, and that is myself. Then it doesn't help if others use their mouths and say that smoking is not good. Because then you just want to smoke even more.”

Some used to tell friends and family about quitting as a way of building a commitment to quitting. Many participants also expressed a strong belief that they themselves knew how to quit smoking and that the treatment offered was not decisive for the result:

Anna, A: “… I know I can do it myself. If I am struggling to quit smoking or as some people certainly do, then I should do it (referring to go to the HLC). But I know how it works and how it should go, no I just do not quite see the point that I should go there and yes, yes in a way we all know.”

Among former smokers, important facilitators appeared to be making the decision to stop smoking and finding their own mental strategies to deal with the cravings:

Lisa, A: “So there is no one who says that I cannot go and buy myself a pack of tobacco other than my head. I have come this far so I cannot. But it's the mindset that okay, do you have the same desire to smoke in 5 min. Yes, well, okay, but then it is gone.”

Among current smokers, most of them were motivated to quit or were in the process of quitting, and several explained that within the given time, they would quit smoking completely. They understood that smoking damages their health and that quitting smoking could potentially help improve their quality of life or help them achieve future dreams, e.g., engaging in activities requiring better physical health. Participants also reported feeling bad about smoking, constantly being reminded that smoking is not good:

Peter, R: “… I do have a bad conscience toward myself and the whole system every time I take a smoke. // After all I know I should, I have stopped with things before, and I know that there is only one way to stop and that is to stop.”

##### 3.1.1.2. Concerns about their health condition

All participants mentioned health-related topics as a facilitator in changing their smoking habits. Concerns for health and the hope for extended life were, for many participants, a strong motivation for quitting smoking regardless of smoking status:

Henrik, A: “I'm starting get old now, it's probably easier to think a little more about health than you did when you were younger. I have turned 72, so then you must start grabbing the sails, or that's what I think.”

Oscar, S: “What creates motivation is that I do not feel in as good shape as I was, I feel more tired than I was before when I did not smoke. So that's probably the strongest motivation, that you should get back in shape and do something other than just smoke.”

Their recent CVD event and information received about the relation between smoking and health were described as major facilitators by most of them. For some, the link was self-evident, whereas others gradually realized the consequences of their smoking:

Henrik, A: “It was the operation that started the process for me, yes I try to do things a little different than before. I was a little scared as I said, because of these blood vessels which gave me painful legs.”

Peter, R: “What had the strongest effect on me, was actually the nurses who cared for me and who told me that you just have to do this, you have to stop smoking, because we see what happens. Those who do not quit, they return. So, then I trusted them because they are in the middle of it. At least it made a strong impression, because I do know that they are in the front-line receiving patients.”

For many, achieving dreams which depend on better health was seen as another facilitator to quit smoking:

Hans, R: “If my health improves, everything else will improve. Then maybe I can start working a little too. That is a big dream.”

Thomas, A: “I do have wishes, and I'm beginning to realize that I have been out and about a lot, I have done a lot of positive things, and I would like to do it, some of it again. And if I am going to be able to do that, I have to live differently.”

##### 3.1.1.3. Financial reasons as a motivating factor

Several participants also pointed out the costs of smoking and a desire for an improved economy as a facilitator of smoking cessation.

Lisa, A: “Another thing is that you cannot afford to live in Norway on a minimum pension and smoke, it's that simple. No one can afford that.”

Thomas, A: “Every time I might have bought cigarettes, I could put that money aside and then I could use it to buy crypto. And then I did not have the conscience to do both or could not afford it for that matter // and what I earn there I will use to buy me a boat, and then I'm going to sail to (place).”

Another participant felt that he was wasting his money away:

Oscar, S: “And now both my wife and I smoke, and 1 year we calculated this, last time when I stopped smoking, and then we spent 70 000 NOK a year on tobacco, and that is 70 000 NOK thrown out of the window.”

##### 3.1.1.4. Behavioral strategies facilitating cessation

All participants talked about actions or events that made them forget or avoid smoking, described as being conscious or unconscious strategies. They discussed the importance of having found their own ways to help them to cease or reduce smoking. Many had also received advice from acquaintances who had quit earlier or followed advice from the treatment, including simple distractions and actively changing daily routines. Some appeared to become aware of such strategies through reflection during the interview:

Hans, R: “I mean then it's much better to maybe have put on a nicotine patch instead, or gone to chop a little wood or, really you have to come up with something instead of sitting and thinking about that craving.”

Oscar, S: “When the weather gets warmer, then it is possible to go out. I hope we can pack and travel again when some time has passed, and then the motivation will be completely different, when I swim half an hour/hour every single morning // and then you simply have no need for that cigarette.”

A particular strategy, which has been helpful to many, was the understanding that the craving to smoke was reduced when removing the tobacco from everyday life or in certain situations:

Peter, R: “I have deliberately chosen to leave the package at home if I am going down in the boat, for example. Because if I bring the tobacco, I smoke, but if I do not have it, I cannot smoke. I have done several such initiatives then. If I leave it without knowing it, then it is not a crisis, I do not turn onto the road and drive back to pick it up.”

Another strategy has been restructuring everyday life in the form of breaking old habits, or finding replacements for smoking, both to prevent and manage the urge to smoke:

Thomas, A: “When I'm at work and there is little to do, then it might happen that I want to smoke. Then I try to implement other things, like sitting down with a homemade excel sheet, I think that's cool.”

Anna, A: “Instead of waking up and have a cup of coffee and a cigarette, now I take slices of bread with cheese and ham or something like that, or I make waffles or pancakes.”

Others have found that smoking cessation medication has reduced the urge to smoke, which many mentioned as difficult to overcome:

Lisa, A: “But I started 14 days before with the tablets. And I have to say that on that particular date, it was supposed to be the last cigarette, but the cigarette tasted bad. Those tablets must have done something because the cigarette tasted worse and worse. I did not finish the last cigarette, but I was supposed to smoke because tomorrow I should not, but I did not do it, because it did not taste good.”

#### 3.1.2. Contextual factors

##### 3.1.2.1. Influence from social environments

For several participants, social settings could facilitate smoking reduction or cessation. Participants chose not to smoke in social settings with non-smokers, either because they did not want to expose them to smoke or because they forgot about smoking in such situations.

Mike, R: “The people I work with don't smoke, so it's a plus to start working again. So that is nice, then you'll be taken care of. Yes, so it's a big advantage // before there were such smoking points in a way, yes there were several different places, so I had to go far away, then I'm spared from that. Because there is no one who does it, I can't go alone to a corner!”

Additional facilitators of cessation mentioned being aware that their surroundings would like them to quit smoking; some received active support from friends and family, and some wanted to avoid the smell of smoking. For those who had a life partner who smoked, the thought of quitting smoking together was a strong facilitator:

Emilia, A: “The most important reason was that smoking was not a good thing, also I listened to the cough of my partner who wanted me to quit. Yes, shall we stop then? We can do it, right?”

##### 3.1.2.2. Helpful components of cessation treatment

The cessation treatment encompassed several facilitators to cessation mentioned by the participants. First, a sense of fellowship related to cessation, especially with like-minded people who were in the same situation, was motivating for several participants. Helpful suggestions and conversations highlighted as the most effective were those with other smokers who were also in the process of quitting smoking:

Lisa, A: “But it helped a lot, that an hour every Tuesday, to hear what the others were struggling with and to be able to exchange feelings and how it went, all six then. // Because then one had experienced it in one way, and another could comment; maybe if you do this and that it will get better, and yes, so we came up with such advice for each other.”

Those who had relapsed or not quit smoking, and who did not use any therapy provided in the RCT, also expressed their need for a discussion group with other smokers if they should participate in future cessation treatment:

Peter, R: “It was really up to me then to quit smoking, but if we had maybe, I do not know what to say, a group, yes just like an AA meeting or something, where there are others with the same problem, because then there will be talk about it, like I did it that way and did you do it too.”

Even though many expressed positive experiences from the follow-up in the RCT, there were few answers regarding what they found specifically helpful about the treatment. Some stated that they would not have contacted the HLC by themselves without the nurse making the appointment for them. Many participants pointed out that they had appreciated the attention, care, and supporting relationships with healthcare professionals:

Henrik, A: “I think I have received pretty good help as far as I saw it // but I think it's nice to have someone to talk to, I think for example that we have talked together now is positive for me, because then there are people around who are interested in whether you quit or not quit, that is perhaps what is important, that you feel that you are not completely alone.”

Participants who did not want to take on-board the full consequences of smoking talked about good information and strong impressions from health professionals who opened their eyes and that this has helped the participants to change their perspective:

Mike, R: “I have known those consequences all the way. But when you are told by strangers clearly and distinctly and they show you what's going on, then it's a little wake-up. Even though I'm not stupid, I know what's going on anyway. Yes, it's a little painful. // I remember those conversations that this and that happens, and it is pretty clear when you think about it yourself. But you do not think about that at all when you smoke, until you somehow get it served.”

Some participants highlighted the importance of good relationships with their therapists as a motivation to quit smoking, even if smoking cessation was not the focus of their conversations:

Thomas, A: “There is a psychologist on the rehabilitation center, where we have talked about accepting that you have been ill and picking out the cards that have gone wrong and using the cards that are left. You also have to try to make the most of the remaining cards and do what you want, because you live now.”

One of the participants found good support from his general practitioner:

Oscar, S: “We do not talk much about the smoke, we talk about health, but inside there is a bit about the smoke, so he will probably give me good help without him talking about it. He gives me such good support without talking about it. What it does is that I feel very safe, that I trust what he says. I think he is very good at listening, and he asks thousands of questions I was about to say, he is very good at asking, yes I have confidence in him.”

#### 3.1.3. Summary—Facilitators

All patients mentioned several factors as crucial for being able to reduce or quit smoking, and these often strengthened the intrinsic motivation over time. Motivational factors most often mentioned were improved health, finances, and environmental influences. Several also expressed a desire to quit smoking, and for some, the last hospitalization was the final push they needed to quit completely.

### 3.2. Barriers to smoking cessation

#### 3.2.1. Individual factors

##### 3.2.1.1. Upside of smoking

Perceived benefits from smoking were, for many participants, a barrier to smoking cessation. Smoking represented many comforts and support, reward, and enjoyment:

Henrik, A: “I have had periods myself where I kind of halfway at least tried to quit smoking, and it is so typical, I have a boat that I enjoy in the summer and I remember I had a period in the spring where I stopped smoking for a while and it lasted until I got the boat on the water and thought it was so cozy to smoke again. And then I almost fell back into the old track. So, it was kind of a bit like that, always been a bit attached to the situation sort of.”

Lisa, A: “I've tried to quit before, and I have not succeeded, I got upset! Just like you lose a good friend.”

Smoking was described as an important part of participants' life for years, becoming a natural part of their daily life, routines, and social interactions, and some could not imagine how they would fill the void if they stopped smoking:

Hans, R: “But what should one do instead of smoking then? So, I should go out and sit knitting then? It's nothing special to ride the bike and meet the boys and then bring out the knitting. Then they would have died of laughter to put it that way. No, but that was a bad example, but what are you going to use your fingers for then, when you are not going to smoke?”

Several also mentioned that it is difficult to quit when feeling lonely or bored, as one quickly resorted to smoking when one could not find anything else to fill the time with.

Oscar, S: “I left for South-Europe, and then I did not smoke much, but then we came home in December, and I started to smoke again, when we came home to the cold and sat inside.”

##### 3.2.1.2. Difficult life situations

Many participants described challenges in life as an important barrier, causing a decrease in motivation for taking the final step toward quitting smoking. Participants talked especially about health problems but also factors related to work, family, and stressful periods with psychosocial stress as reasons why they had not stopped smoking, or as possible causes of relapse.

Oscar, S: “My wife lost her son, so I'm a bit out of sorts. // So now I smoke a bit, I must admit, because life goes up and down all the time. So, the reason I haven't quit is that now there is too much going on.”

Kristian, S: “I have been to several funerals in a short time. It's about finding the right time to quit, and for me it hasn't been the right moment. My wife also fell ill. Everything is a little uneasy, too much is going on at the same time, as I think there are emotions and the psyche that controls a lot of my smoking and smoking habits.”

Others said that their poor health situation had led to negative spirals of thoughts and discouragement, leading to continuing smoking:

Peter, R: “There is also something about the fact that I have thought a lot about this life of mine, and I wonder, does it have something to say? That I am dying now? My God I have several health conditions, yes, I must have been a little self-pitying and got into some stupid things that I thought, is it worth it (to quit smoking)?”

For many, smoking was a support and a source of emotional regulation during difficult times, thus reducing motivation to quit:

Hans, R: “After two and a half months in the hospital, it was just before that, I must say, it was just before I ended up behind a corner and cried a little. And this is not me at all. But then it felt good with a cigarette, I must say. It was good to sit there and feel that you became calm inside. It may well be that it's a bit like, I do not remember what you call it, but that it's a little imagination. Belief moves mountains, right.”

Troubled life situations were by many participants also used as an excuse to why they had not quit smoking yet:

Oscar, S: “When things go up and down in life, it's hard. Now I probably use it as an excuse I think, to be honest.”

Peter, R: “It's unconscious thoughts like, there's a reason why you smoke right, then you're more physically and psychologically addicted to it, but we're simply justifying it, because I feel soooo bad, you know, right. One becomes an expert, experts at it, self-pity.”

##### 3.2.1.3. Addiction

One factor that made it difficult for participants to quit was addiction, which often manifested as cravings or long-term habits. Participants talked about a craving so strong that they could not resist and that this was a major cause of failed quitting attempts:

Hans, R: “The craving takes over right, that's when I start to think that the smoke decides. Because when the urge comes, then in the end you cannot resist. Then you must go out and smoke right. And then I think it's a bit of a loss. Because I'm a bit like that (indistinct) type, and I manage all other things well in life. And been through a lot of serious health conditions without having said anything negative. So, I lived my life to put it that way. And I managed to get through, but I'm not able to stop smoking. Then I almost get annoyed.”

Everyone associated smoking with habits, of which some were more difficult to get rid of than others.

Henrik, A: “I realized myself that it was a kind of a ritual really you can say // yes then I think of before you take a cup of coffee in the morning and go to the bathroom and such, then it became easy to take a cigarette. And especially after dinner, also when you should have an especially good time or something like that, then it was easy to crawl to the cigarette.”

#### 3.2.2. Contextual factors

##### 3.2.2.1. Contact with other smokers

Social settings with the presence of other smokers were described as difficult situations keeping abstinent from cigarettes, and several said that such social influence was the cause of previous relapses. One participant had been abstinent for 13 years before a weak moment got him back on old tracks:

Oscar, S: “I was alone and refurbished the house, and then people kept coming by, when I was alone, and I had to sit down and talk a little. Then the pack of cigarettes was introduced with the question, are you not going to have a cigarette? No thanks, and they said it twice and then you smoked the third time right. So, I wasn't stubborn enough, also that I might have been a little tired and stuff, had been working all the time, and then when someone came and kept me company, I couldn't resist to smoke.”

For many, tobacco had been a natural part of social meetings, giving a sense of fellowship with other smokers, whether it was a shared coffee on the balcony or at larger events:

Lisa, A: “I've been in two or three parties this summer with people I don't know, or I know some, but not absolutely all. Now I'm a person who goes around and talks to people, but otherwise you go out and have a cigarette, and then you've already met maybe 10–15 people. There are a lot of people outside smoking and when you come back in, you have some points of reference. And that is why I say that it is much more social to smoke. So, get me right, it's easier to get in touch with people because you're doing the same thing. And you don't talk about the smoke either, but you stand there and wonder where you're from, and who you know here in the company, so that kind of talk.”

Some also had regular smoking routines that they shared with their partners or work colleagues, making it difficult to quit smoking:

Peter, R: “If there are several, we‘ll have a cigarette together, especially on the type of job I have. // I work with drug-addicted youth, and it is a very nice way to get in touch with especially the new ones then, which is a bit like a new smoker, because something is going to replace what they have stopped with.”

##### 3.2.2.2. Lack of support and understanding among healthcare professionals

A barrier to several participants was experiences of moralizing healthcare professionals, which created resistance to following instructions and quitting smoking. Participants brought up feelings that they were not treated as equals in meetings with healthcare personnel and that they were met with a lack of understanding, condescending attitudes, or moralizing behaviors. For some, this has resulted in them refusing treatment offers from the RCT.

Hans, R: “There was also a focus on smoking cessation at the rehabilitation center. But it was nothing, it was really just a hassle. It was more like bugging. It was like from above and down, there it was distrusted and disliked smoking. And then I become such a kindergarten I mean, if you talk from above and down to me then I become a bit like a kindergarten child. Then I do not bother to listen more to you. You do not need to take that morale in front of me, because I know this is not good, right. So, talk to me, not down to me somehow. Those are two slightly different things.”

#### 3.2.3. Summary—Barriers

Barriers to smoking cessation, combined with the perceived benefits of smoking, including emotional regulation and addiction through habits and cravings, made the participant's motivation vulnerable to difficult life situations and other excuses for continuing smoking. Participants also talked about how health professionals have reduced their motivation instead of functioning as support toward smoking cessation.

## 4. Discussion

To the best of our knowledge, this is the first study analyzing facilitators and barriers to smoking cessation in a CVD population who have been hospitalized for an acute CVD event, a particular teachable moment for facilitating cessation. In particular, all study participants had also been offered recommended in-hospital cessation management and close and continued outpatient follow-up care with access to free cessation aids.

Our results show that participants have several facilitators weighted against barriers, which seem to create an ambivalence toward quitting smoking, even though their motivational score was generally high. Similar results have been found in previous studies, but not for a CVD population or in this specific setting.

Throughout the process of the indicative analysis, it became apparent that our results map well onto the theory of planned behavior (TPB) (Ajzen, 1991). We have, therefore, discussed the results using this theory as a conceptual framework for ease of interpretation.

### 4.1. Attitude toward the behavior

According to the TPB (Ajzen, 1991), the key component to carrying out a behavior is the intention to engage in it, which indicates how much effort at an individual level is required to be exerted to perform the behavior. The intention is determined by three psychosocial determinants: attitudes toward the behavior, subjective norms, and perceived behavioral control, which act as motivating factors that will influence the behavior (Ajzen, 1991).

In this context, the attitude refers to the evaluation of quitting smoking as favorable or unfavorable. Similar to the results of other qualitative studies, a positive attitude toward cessation appears to be mainly driven by concerns about the health situation, as well as financial incentives (Kerr et al., 2006, 2013; Clancy et al., 2013; Buczkowski et al., 2014). This attitude seems to be strengthened through the influence of social circles, family, and health personnel. On the contrary, positive attitudes conflict with perceived barriers to cessation. Barriers found of greatest importance are the upsides of smoking, tobacco as an emotional regulator in stressful situations, and addiction through cravings and habits. Although all participants had a goal of quitting smoking, the barriers outweighed the facilitators for the persistent smokers, interfering with their inner motivation. Our results indicate that, in high-risk situations, immediate satisfaction will become more important than the long-term goal because they use tobacco to modulate stress and emotions during difficult periods or, in learned situations, are unable to resist the temptation to smoke. In this way, the balance between facilitators and barriers can be seen as a dynamic process, where the balance will tip back and forth in a short-term perspective. In turn, this may explain why some are unable to quit or end up relapsing after a short time despite having a future goal of quitting smoking, despite a positive attitude toward smoking behavior.

### 4.2. Subjective norms

Subjective norms in the TPB refer to the social environment that influences the behavioral intention through the perceived social pressure to engage in the behavior (Ajzen, 1991). In the current study, former smokers missed smoking in social gatherings, while current smokers are easily tempted to have a cigarette in smoking environments where it is socially acceptable to smoke. Social influence plays a particular role in terms of relapse. Former and current smokers blamed social occasions with other smokers as an explanation for previous and current relapses, as found in other qualitative studies (Buczkowski et al., 2014, 2021). This may be due to the lack of social support and exposure to smoking cues (Shiffman et al., 1996, 2002; Hitchman et al., 2014; Cambron et al., 2020), hence reflecting the classic learning models of addiction (Shadel et al., 2000). Our study population is mainly characterized by people with low educational levels and poor income, a group more likely to smoke compared to those with higher socioeconomic status (Laaksonen et al., 2005; Hiscock et al., 2012). This increases the probability of being part of social circles of smokers, making quitting or remaining abstinent more difficult due to frequent exposure to smoking cues. Similarly, living with a smoker is also found to be a barrier to sustained abstinence and successful quitting attempts (Ferguson et al., 2005; Homish and Leonard, 2005; Lewis et al., 2006).

On the contrary, our results show that support, but also pressure, from healthcare personnel, colleagues, and close relationships have, for many participants, been an additional facilitator for cessation, coincident with other results (Kerr et al., 2006; Medbø et al., 2011; Dieleman et al., 2021). The absence of other smokers in social settings seems to be an effective remedy as this often leads to non-smoking. Finally, conversations with others who have stopped smoking or who were in the same boat regarding thoughts of quitting have had a strong impact. These results highlight the importance of taking the patient's social context into account when promoting smoking cessation.

### 4.3. Perceived behavioral control

An important factor influencing the decision to engage in a behavior, both directly and indirectly, is perceived behavioral control, referring to an individual's expectations regarding performing the behavior based on self-efficacy (Ajzen, 1991). High self-efficacy is a strong predictor of successful cessation including making quit attempts, staying abstinent for a prolonged time, and the likelihood of re-establishing abstinence after relapse (Condiotte and Lichtenstein, 1981; Stuart et al., 1994; Dornelas et al., 2000). Most of the participants expressed an intrinsic motivation to quit smoking, which seems to include the following three factors: autonomy, commitment, and self-efficacy. Some also stated that they knew how and were able to quit smoking by themselves. However, it appeared that current smokers were ambivalent toward quitting, contradicting themselves, saying they were motivated, able, and committed to quitting, but also providing several excuses as to why they had not done so yet. Perceived behavioral control is determined by smokers' total set of control beliefs. Persistent smokers expressed a high self-efficacy to stop smoking, but the coping belief seems to be situational. Factors such as smoking social circles and challenging life situations may reduce participants' self-efficacy if they do not have coping strategies to deal with the smoking cues. The preference for quitting unassisted is in line with the findings of other studies, as well as the fact, for many, it is their own willpower that is needed to quit (Smith et al., 2015; Gravely et al., 2021). Our results show that some smokers nevertheless may benefit from follow-up in line with patients' desire for acceptance, autonomy, and a non-judgmental approach.

### 4.4. Clinical implications

In this study, most of the participants focused on their autonomy in the process toward cessation. It was critical to them that quitting smoking had to be done on their own terms. The feeling of control over one's own life and choices was an important source of motivation and, thus, has implications for motivation for undertaking treatment and the type of treatment. Feeling supported was highlighted as positive in meetings with healthcare professionals and most of the participants felt seen and treated respectfully. This contrasted with their previous experiences of moralizing health professionals who tried to convince or force them to quit smoking, which was known to raise resistance toward change (Evans-Polce et al., 2015). This may also explain why some participants were eager to quit unassisted. Motivational interviewing (MI), a counseling method for eliciting motivation and supporting behavioral change in an exploratory, supportive, and non-confrontational manner, aligns well with the needs of the patients (Miller and Rollnick, 2013) and is highly recommended to improve motivation for behavioral change (Piepoli et al., 2016). A non-judgmental approach such as MI allows the individual to freely reflect on his/her ambivalence and to explore the benefits and costs of continuing smoking, which also support the need for autonomy. The empathic approach is important for the therapeutic relationship and the individual's relatedness. MI also has the potential to build inner motivation for smoking cessation through enhanced perceived importance and self-efficacy, which is comparable with the TPB in the way of supporting competence (Miller and Rollnick, 2013). Thus, MI may be particularly well suited to support smoking cessation in patients admitted with CVD.

Furthermore, current smokers also expressed an ambivalence toward quitting smoking, expressing that “it was not the right time to quit” due to difficult and stressful life situations, which is possibly related to a lack of coping and emotion regulation strategies other than the perceived benefits of smoking. These results emphasize the importance of eliciting stressful life events or circumstances for those who do not manage to quit smoking, despite being highly motivated.

In this study, half of the participants managed to sustain abstinence for 6 months, while the rest either relapsed or never quit smoking. The persistent smokers were nevertheless motivated to quit and expressed an intention to cease smoking in near future. Thus, it is reasonable to believe that repeated information and extended follow-up with behavioral support and cessation drugs could enhance the probability of successful cessation. An acute hospitalization is also a teachable moment to introduce help for smoking cessation (Rice et al., 2017). However, consistent results from a Cochrane review exploring interventions for smoking cessation found that cessation counseling interventions initiated at the hospital increased cessation rates, but only if it included follow-up for at least 1 month after discharge (Rigotti et al., 2012). Follow-up by regular conversations, either digitally, through phone, or at the HLC, and the fact that participants were directly referred to HLC were highlighted as positive and emphasized the importance of scheduling follow-up appointments before hospital discharge. Many also profited from or asked for groups with other smokers as an opportunity to share experiences and advice with like-minded people instead of being informed by people who have never smoked before. Non-specific factors such as attention, trust, and support seem to be equally important. Consequently, our results indicate that follow-up should be individualized based on the smokers' stage in the process of quitting and smokers should be offered to participate in group conversations with other smokers.

## 5. Strengths and limitations

Strengths of our study were as follows: We have included participants from the intervention arm of a cessation RCT conducted in a routine clinical setting among a high-risk group with established CVD, who have substantial health benefits from quitting smoking. Participants had varying grades of nicotine addiction, and there was a 50/50 balance between those who had managed to quit and those who still smoked. Given the lack of pre-existing knowledge of barriers and facilitators in this patient group and the high rates of non-cessation, a qualitative design was deemed appropriate.

There are some limitations. In total, 10 out of 25 potentially eligible patients attended the qualitative study. Unfortunately, we did not register the reason why patients declined participation. Therefore, we do not know if all facilitators and barriers to cessation representatives for this population have been identified. Furthermore, because of a small sample size, it is difficult to know whether the participants' views are representative of all smokers with CVD. All participants in this qualitative study were above-average motivated, which is a limitation for generalizing the results. Further studies should explore facilitators and barriers among smokers less motivated.

Some topics of potential importance, including the influence of society or the participants' feelings related to being a smoker, have not been covered in the interviews. Even though the interviewer tried to be neutral and non-judgmental, it cannot be ruled out that some participants felt uncomfortable or judged and, therefore, adjusted their answers. Finally, telephone interviews are considered a satisfactory method of obtaining data in qualitative interviews (Farooq and De Villiers, 2017), but the interviewer may have lost some information due to the lack of visual language, which, in turn, might have prompted further questions.

## 6. Conclusion

This study explored perceived facilitators and barriers to cessation in smokers with CVD that had been offered medical and behavioral follow-up care. Results indicate that, despite overall high motivation to quit for the long term, some participants do not have the attitude or behavioral control to cease smoking in immediate future.

The clinical understanding of this analysis is that smokers motivated to cease smoking should receive a communication approach addressing ambivalence, motivation, and level of self-efficacy in an exploring and non-judgmental way, making MI a potentially suitable method. The upsides of smoking, particularly coping with stressful events, should be explored further to help patients find a replacement or alternative coping strategies. Furthermore, our results emphasize the importance of addressing the smoking environment as a potential barrier to achieving successful cessation. Finally, many smokers prefer talking and sharing advice with other smokers, indicating that focus groups for smokers ready to quit could be implemented. Smokers move through the stages toward cessation at different speeds; thus, treatment and follow-up programs should therefore be tailored to the needs of each individual.

## Data availability statement

The datasets presented in this article are not readily available due to data sharing restrictions in Norway. Further enquiries should be directed to the corresponding author.

## Ethics statement

The studies involving human participants were reviewed and approved by REC South-East B 461524. The patients/participants provided their written informed consent to participate in this study.

## Author contributions

JM and TD contributed to the idea and design of the study. VG contributed to the data collection and was responsible for the first draft of the manuscript. VG, TD, HL, and JM contributed to data analysis and interpretations. All authors contributed significantly to the final version of the manuscript.
