# Supplementary material for: Barriers and facilitators for smoking cessation in chronic smokers with atherosclerotic cardiovascular disease enrolled in a randomized intervention trial: A qualitative study
Source: Front Psychol. 2023 Mar 22;14:1060701. doi: 10.3389/fpsyg.2023.1060701 (PMC10074255; doi:10.3389/fpsyg.2023.1060701)
Supplement: Supplementary file 1 [file Data_Sheet_1.PDF]

INTERVIEW GUIDE - QUALITATIVE STUDY OF THE RCT SMOKING CESSATION INTERVENTION

We want to ask you some questions about your experiences participating in the smoking cessation study and help us understand why some patients stop smoking, while others continue or relapse.

1. Opening questions:
   - What is your smoking status?
   - Have you participated in the Healthy Life Center?
   - Have you used any smoking cessation drug?
2. What do you think are the reasons why you have succeeded/failed/relapsed in smoking cessation?
3. What were the decisive factors?
4. Could you tell about the importance of the components of the smoking cessation intervention, including the counselling you received from the nurse in the hospital, participation in the Healthy Life Center and use of smoking cessation drugs, for the outcome?
   - How did you experience the follow-up you received?
   - What, if anything, do you think was beneficial about the follow-up interventions you received?
   - What, if anything, was not beneficial?
5. What expectations did you have before you started the smoking cessation intervention and to what extent were they fulfilled by the intervention?
6. How motivated were you to quit smoking before you entered the smoking cessation intervention? (What strengthened/reduced the motivation?)
7. Did you take any action on your own to succeed quitting smoking? Was this related to the smoking follow-up you received?

8. To those who have quit smoking:
   - Can you tell me about the period when you quit?
   - Could you describe the days as a non-smoker? Do you miss smoking now? Do you still feel the urge to smoke? Describe.
   - What did you learn, if anything, in the smoking cessation program that helped you stay smoke-free?

- What will help you to maintain the status of being smoke-free?
- If later relapse later, would you seek help again? Why/why not?

9. To those who relapsed:
   - How long did you stay you smoke-free? Can you tell me about this period?
   - What was/were the main reason(s) why you decided to start smoking again?
   - Do you have concrete plans to seek help again? If so, what kind of help? Why/why not?
10. To those who are still daily smokers:
    - Were you at any point during or after your hospitalization for heart disease ready to quit smoking? Was the motivation higher at any point?
    - What do you think it will take for you to quit smoking? More follow-up from health personnel? Other?
    - Do you have concrete plans to quit smoking today?
